# *FCGR2C* Polymorphisms Associated with HIV-1 Vaccine Protection Are Linked to Altered Gene Expression of Fc-γ Receptors in Human B Cells

**DOI:** 10.1371/journal.pone.0152425

**Published:** 2016-03-25

**Authors:** Xinxia Peng, Shuying S. Li, Peter B. Gilbert, Daniel E. Geraghty, Michael G. Katze

**Affiliations:** 1 Department of Microbiology, School of Medicine, University of Washington, Seattle, Washington, United States of America; 2 Department of Biostatistics, Bioinformatics, and Epidemiology, Vaccine and Infectious Disease Division, Fred Hutchinson Cancer Research Center, Seattle, Washington, United States of America; 3 Clinical Research Division, Fred Hutchinson Cancer Research Center, Seattle, Washington, United States of America; University of Alabama at Birmingham, UNITED STATES

## Abstract

The phase III Thai RV144 vaccine trial showed an estimated vaccine efficacy (VE) to prevent HIV-1 infection of 31.2%, which has motivated the search for immune correlates of vaccine protection. In a recent report, several single nucleotide polymorphisms (SNPs) in *FCGR2C* were identified to associate with the level of VE in the RV144 trial. To investigate the functional significance of these SNPs, we utilized a large scale B cell RNA sequencing database of 462 individuals from the 1000 Genomes Project to examine associations between *FCGR2C* SNPs and gene expression. We found that the *FCGR2C* SNPs that associated with vaccine efficacy in RV144 also strongly associated with the expression of *FCGR2A/C* and one of them also associated with the expression of Fc receptor-like A (*FCRLA*), another Fc-γ receptor (FcγR) gene that was not examined in the previous report. These results suggest that the expression of FcγR genes is influenced by these SNPs either directly or in linkage with other causal variants. More importantly, these results motivate further investigations into the potential for a causal association of expression and alternative splicing of *FCGR2C* and other FcγR genes with the HIV-1 vaccine protection in the RV144 trial and other similar studies.

## Introduction

The RV144 preventive HIV-1 vaccine efficacy trial tested the ALVAC-HIV-1 plus gp120 AIDSVAX B/E vaccine regimen in Thailand and demonstrated an estimated vaccine efficacy (VE) of 31.2% for prevention of HIV-1 infection [1]. Several follow-up studies were conducted to search for the correlates of risk for HIV-1 infections and to investigate the predictors and the mechanisms of the vaccine protection [2–6]. Among them, Li et al reported that *FCGR2C* (Fc fragment of IgG, low affinity IIc, receptor for (CD32)) polymorphisms associated with the HIV-1 vaccine protection [2]. By sequencing the external protein-coding domains and the transmembrane regions of the five low-affinity FcγR genes (*FCGR2A*, *FCGR2B*, *FCGR2C*, *FCGR3A*, *FCGR3B*), the study found that one *FCGR2C* tag SNP (rs114945036) in linkage disequilibrium with 3 other *FCGR2C* SNPs (rs138747765, rs78603008, and rs373013207) was associated with VE against HIV-1 subtype CRF01_AE. Individuals carrying CC had an estimated VE against HIV-1 subtype CRF01_AE with lysine at position 169 (169K) of 15%, while individuals carrying CT or TT exhibited a VE against CRF01_AE 169K HIV-1 of 91%. For VE against any HIV-1 subtypes, the individuals carrying CC had VE = 11% compared to VE = 64% for the individuals carrying CT or TT. Overall, the study provided a strong evidence for a role of FcγRs and Fc-mediated Ab function in the protection conferred by the RV144 vaccine regimen against HIV-1 acquisition. However, the functional significance of these associations, such as a causal relationship to alternative splicing or other changes in FcγR expression patterns or levels under different conditions, was not established [2].

## Materials and Methods

RNA-seq read mapping, genotypes, expression quantification and eQTL mapping results reported by [7] were downloaded from EBI ArrayExpress (accession E-GEUV-1). As detailed in [7], the mRNA quantification was calculated at different levels. For exon quantification, overlapping exons of a gene were first merged into meta-exons. Transcripts and splice junctions were quantified by the Flux Capacitor approach [8]. Gene quantification was calculated as the sum of all transcript RPKMs (reads per kilobase per million) for each gene. Before eQTL analysis, expression quantifications were normalized by PEER correction [9] and transformed into standard normal distribution. eQTLs were mapped using a linear model in Matrix eQTL [10] and permutations were used for FDR estimation.

For FcγR genes with updated RefSeq annotation, the number of reads uniquely mapped to each gene in each sample was counted using HTSeq [11], and the same read mapping as in [7] was used. The RefSeq annotation was downloaded from UCSC genome browser (https://genome.ucsc.edu, accessed on July 1, 2015). The pairwise LD (both *D*’ and *r*^2^) between the SNP rs114945036 and other SNPs was individually estimated based on the genotypes of the 373 individuals from EUR using PLINK [12]. VCF files were first converted into PLINK format using VCFtools [13]. The similarities between RefSeq annotated *FCGR2A* and *FCGR2C* transcript sequences were calculated using Exonerate [14]. Additional statistical analyses including boxplot and Pearson correlation were performed using R (https://www.r-project.org).

## Results

### *FCGR2C* polymorphisms associate with FcγR gene expression in human B cells

**Fig 1:**
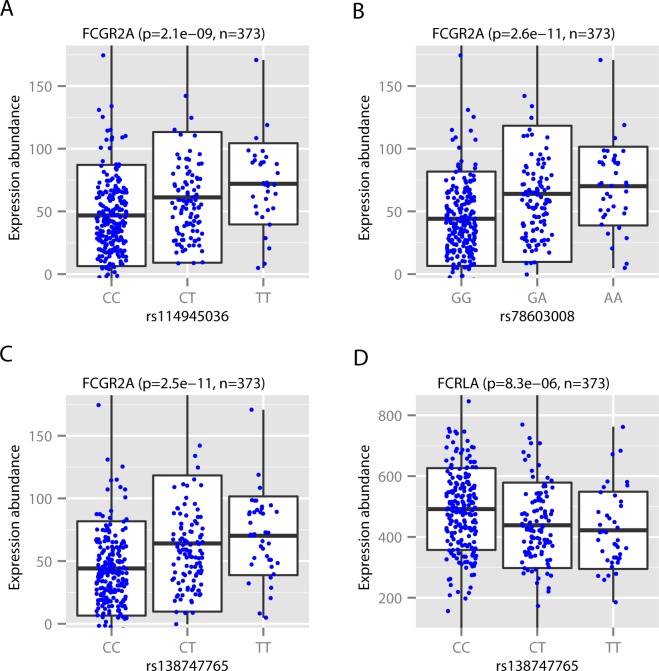
*FCGR2C* polymorphisms associate with FcγR gene expression in B cells in the European (EUR) population. A. The boxplot shows the distribution of the expression (y-axis) of the last exon (hg19, chr1: 161487765–161489358) of *FCGR2A* in B cells from the 373 EUR individuals as quantified in [7], stratified by the genotypes (x-axis) of the SNP rs114945036. Individual expression levels are (horizontal line = median; bottom and top of box = 25^th^ and 75^th^ percentile). Expression in individuals is shown in blue dots. The significance of the association is indicated immediately above, which were mapped in [7] using a linear model implemented in Matrix eQTL [10]. B. Similar as A, for the SNP rs78603008. C. Similar as A, for the SNP rs138747765. D. Similar as A, for the SNP rs138747765 and the expression of the third exon (hg19, chr1:161680550–161680702) of *FCRLA* (Fc receptor-like A) in B cells.

To investigate the functional consequence of the relevant genetic variants [2], we analyzed a published large-scale B cell RNA sequencing dataset from the 1000 Genomes Project [7]. Lappalainen et al performed mRNA sequencing on lymphoblastoid cell line samples of 462 individuals in an attempt to uncover functional variants in humans at the genome scale [7]. They mapped *cis-* quantitative trait loci (QTLs) to transcriptome traits of protein coding genes and miRNA genes, separately in the European (EUR, n = 373) and Yoruba (YRI, n = 89) populations. We reasoned that this integrated transcriptome and genome sequencing dataset would allow us to investigate in detail the impact of individual SNPs [2] on gene expression. Interestingly, we found that three of the four SNPs described in [2], rs114945036, rs138747765 and rs78603008, were significantly associated (FDR < 0.05 by Benjamini-Hochberg method [15]) with the expression level of *FCGR2A* in the EUR populations (Fig 1). The reason that these significant associations were found only in the EUR population may have been due to the larger sample size (373 EUR vs. 89 YRI) in the eQTL dataset and a relatively higher minor allele frequency for all three SNPs in the EUR population (S1 Fig). Especially, all three SNPs were associated with changes in the expression of the last exon of *FCGR2A*. These three SNPs were in a complete linkage disequilibrium (LD) in the original RV144 study [2], and they were also in a strong LD in EUR (rs114945036 vs. rs138747765: *D’* = 1, *r*^2^ = 0.83; rs114945036 vs. rs78603008: *D’* = 1, *r*^2^ = 0.83, where *D’* is a standard measure of LD).

### *FCGR2C* polymorphisms likely directly associate with *FCGR2C* gene expression in human B cells

Since these three SNPs are relatively distant from *FCGR2A* (over 84 kb away from its transcriptional start site), we investigated if there were any functional variants which were not genotyped in [2] but were in linkage with these three *FCGR2C* SNPs. A search of all SNPs identified as having a significant association with the *FCGR2A*’s expression in [7] suggested that was not the case. S2 Fig shows that only a few SNPs that are within or near *FCGR2A* passed the significance cutoff of FDR < 0.05, and none of these were in LD with the *FCGR2C* tag SNP rs114945036. In contrast, in addition of the three *FCGR2C* SNPs, we identified several additional 1000 genomes SNPs in and around *FCGR2C* that significantly associated with *FCGR2A* expression, all of which were in linkage with rs114945036 (S1 Table and also see S2 Table). These observations raised the possibility that the newly identified SNPs in the *FCGR2C* region might directly associate with the expression of *FCGR2C* itself.

In order to gain understanding of why *FCGR2C* SNPs were associated with *FCGR2A* expression in this dataset, we undertook a close examination of the Gencode annotation of FcγR genes, on which the analysis published in [7] was based. This analysis discovered confounding errors in the annotation of *FCGR* gene transcripts that may indicate that *FCGR2C* and *FCGR2A* were not distinguished appropriately (Gencode Team, personal communication, July 3, 2015). For example, several transcripts with long introns read through multiple FcγR genes (S2 Fig), which could severely complicate the quantification of *FCGR2C* and other involved FcγR genes. Further, the last exon of *FCGR2C*, which is over 98% identical to the last exon of *FCGR2A* at the nucleotide level, was apparently missing in the Gencode annotation (S2 Fig). These results suggested the association of *FCGR2C* SNPs with the expression of *FCGR2A* could be due to the correlated expression between *FCGR2A* and *FCGR2C* or simply to the inability to distinguish these sequences using short read sequence data. To confirm this possibility, we re-calculated the number of reads uniquely mapped to each FcγR gene, but using the canonical RefSeq annotation of FcγR genes shown in S2 Fig. As expected, S3 Fig shows that the expression of *FCGR2A* and *FCGR2C* was highly correlated (Pearson correlation r = 0.9). Since there is a high sequence identity between *FCGR2A* and *FCGR2C* transcripts (overall ~96% nucleotide identity, S3 Table), it is possible that the correlated expression was driven by certain ambiguities in read assignments, or shared regulatory mechanisms of FcγR gene expression, or both. The former is unlikely because a mappability analysis across the FcγR region showed that there are many regions unique to each of these genes for which their RNA fragments can be unambiguously mapped (data not shown). The complexity of repeating the complete eQTL analysis with updated transcript annotation and the complications of quantifying genes of high sequence identity with short read sequencing data precluded us from directly establishing the association between *FCGR2C* SNPs and its expression. However, it is evident from these results that *FCGR2C* SNPs could also directly associate with the expression of *FCGR2C* itself.

### *FCRLA* polymorphisms associate with *FCRLA* gene expression in human B cells

**Fig 2:**
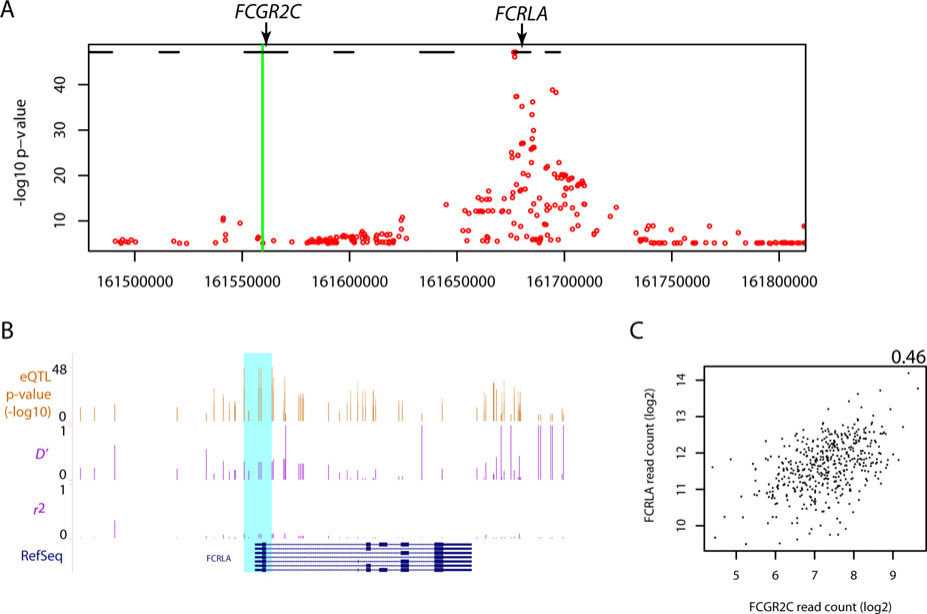
*FCRLA* polymorphisms associate with *FCRLA* expression in human B cells. A. Scatterplot of the eQTL p-values (-log10 scale) for the association of SNPs across the FcγR region with the expression of *FCRLA* as mapped in [7]. For simplicity, for each SNP only the smallest p-value from different levels of expression, i.e. exon, transcript, and gene, is shown. The green vertical bar indicates the location of the *FCGR2C* SNPs identified in [2]. The black horizontal line segments at the top indicate the positions of Refseq annotated FcγR genes, with the locations of *FCGR2C* and *FCRLA* labeled. B. Close view of SNPs around *FCRLA* in UCSC genome browser (hg19, chr1:161,670,571–161,688,007). The top track shows the genomic locations and the association p-values (-log10 scale) for those SNPs that passed the significance cutoff of FDR < 0.05 in their associations with the expression of *FCRLA* at different levels as reported in [7], i.e. exon, transcript, and gene. Highlighted in color cyan are SNPs with smallest p-values (also see S1 Table and S2 Table). The middle two horizontal tracks show the LD between each of the corresponding SNPs shown on top track and the SNP rs114945036. RefSeq gene annotation is shown at the bottom. C. Scatterplot of raw RNA-seq read counts (log2 scale) of RefSeq annotated *FCGR2C* (x-axis) and *FCRLA* (y-axis) in B cells from each of those 462 individuals. The number on the top-right corner shows the Pearson correlation coefficient.

As shown in Fig 1, rs138747765, one of the *FCGR2C* SNPs identified in [2], was also significantly associated with the expression of *FCRLA*, another FcγR gene not examined in the previous report [2]. In addition, a close examination showed that several SNPs in the *FCRLA* gene had much stronger associations with the expression of *FCRLA* (Fig 2A). These SNPs were not in a linkage with the *FCGR2C* SNP rs138747765 (Fig 2B), and their much stronger associations and closer proximity to *FCRLA* is consistent with more direct roles as functional variants for *FCRLA* than rs138747765. Interestingly, the association of rs138747765 with the expression of *FCRLA* could also be partially explained by the correlation between the expression levels of *FCGR2C* and *FCRLA* (Fig 2C). These results suggest that the expression of individual FcγR genes could be influenced by multiple genetic variations within the FcγR region.

## Discussion

Recently, Li et al reported that *FCGR2C* polymorphisms were associated with HIV-1 vaccine protection in the RV144 trial [2], but the functional significance of these polymorphisms was not established. Here, we analyzed a large scale B cell RNA sequencing dataset of 462 individuals, and found that the *FCGR2C* polymorphisms also associated with the expression of FcγR gene *FCGR2A*, and very likely *FCGR2C* itself. In addition, we found that one of these *FCGR2C* SNPs also associated with the expression of *FCRLA*, another FcγR gene in the region, which was not examined in the original report [2]. These results show that the expression of FcγR genes is influenced by these polymorphisms, either directly or in linkage with other causal variants.

This work further supports conclusions from previous studies of genetic correlates of vaccine efficacy in the RV144 trial. Three recent studies collectively pointed to a common mechanistic effect of three different gene groups by acting on antibody responses. HLA class I was implicated by a significant HLA A*02 association with vaccine efficacy [3]. That study suggested that the HLA A*02 allele could have influenced antibody production acting through an exongenous pathway linked to both HLA class I and class II antigen presentation [16]. The study by Prentice et al. [6] directly supported a role of HLA class II allelic variants in modulating HIV-1 vaccine-induced antibody responses. A role for the FcγR proteins in antibody responses is evident, and the present study may point to mechanisms affecting FcγR expression and consequent protein function.

Evidence of genetic variants from three distinct polymorphic gene groups collectively pointing to modulation of vaccine induced antibody responses in a single vaccine trial, highlights a major point relevant to vaccine design–host genetic variation can modulate vaccine efficacy. Genetic variants can affect antigen presentation directly as with MHC class I and class II, or by affecting gene expression levels or splicing patterns, thus affecting derivative protein function through quantitative or qualitative mechanisms, as suggested by this study. While HIV-1 vaccine trials undertaken thus far have each used single source vaccines, consideration of genetic factors may provide a novel avenue for HIV-1 vaccine design. The essential detail of host genetics in the RV144 trial that modulated specific responses and improved vaccine efficacy could not only be used to better understand the mechanisms underlying vaccine efficacy, but also in designing multiple variants of the vaccine itself. Modifications of individual vaccines to produce genetic ‘vaccine variants’ that were matched with knowledge of host genetics, could direct vaccines with the highest efficacy to appropriate genetic subpopulations. Indeed, for HIV-1 and other antigenically variable viruses, such an approach may not only be valid but also necessary.

Clearly, additional studies are necessary to establish the causality of these associations. For example, the gene expression data examined was measured in subjects from the European population and we cannot rule out that other genetic differences between EUR and the Thai population in which RV144 trial was conducted also influence expression levels. However, it has been observed that there is significant sharing of eQTL effects between Asian and European populations overall [17]. It was reported that the expression of a FCGR2C allele (FCGR2C-ORF) in B cells enhances humoral response to immunization in mice and to vaccination in a human anthrax vaccine trial [18], but no data was reported on the frequency of the specific allele in Asian populations and the RV144 population was of Thai origin. A close look at the dbSNP shows 100% of the rs759550223 as T–the other allele variant (FCGR2C-STP) at the same position as FCGR2C-ORF was found in all but one sample in the RV144 samples we analyzed [2]. The low frequency of the FCGR2C-ORF allele in East Asian population was also reported previously [19], suggesting that the associations observed here are less likely driven by this particular allele. It is known that *FCGR2C* is copy number variable, in part due to the *FCGR3A* or *FCGR3B* deletions [19–21]. It will be interesting to investigate if and how the deletion alleles contribute to the observed differences in gene expression, as hemizygotes may appear to be homozygotes for *FCGR2C* SNPs. Since the frequency of these deletion alleles is very low (~5%), this gene dosage effect may be limited.

It is not clear why *FCGR2C* SNPs and not *FCGR2A* polymorphisms were associated with RV144 vaccine efficacy, while changes in expression levels of *FCGR2A* were associated with those SNPs. However, considering the high homology between the *FCGR2A* and *FCGR2C* sequences, the RNA-seq data examined does not allow us to rule out changes in *FCGR2C* expression as well or instead of *FCGR2A*. Also, little is known about the regulation of the FCGR genes and considering their relative locations within the FCGR gene cluster there is no a priori reason to rule out an effect of FCGR2C localized SNPs on *FCGR2A* expression. Another potentially relevant factor is expression changes specific to cell subtypes, as FCGRs exhibit distinct expression patterns in different in lymphocyte subsets [22]. Interestingly, a recent comprehensive immunephenotyping analysis of 78,000 immune traits in 699 female twins reported that the genetic variations in the FcγR locus containing *FCGR2A*, *FCGR2B*, and *FCRLA* had the widest range of impacts on immune cell subset frequency and immune cell-surface protein expression levels [23]. The strongest association for this locus was between a SNP in the *FCGR2A* coding region (rs1801274) and the protein expression of CD32 (FcGR2a and/or FcRG2b) on the surface of inflammatory myeloid dendritic cells. But similar genetic control of CD32 protein expression was not observed in B cells [23]. Though the SNPs examined in [23] were distinct from the *FCGR2C* SNPs studied here, the observations reported together suggest a broad range of impacts on the phenotypes of different leukocyte subsets. At the same time, these results strongly argue that it would be informative to examine if altered expression of *FCGR2C* and other FcγR genes such as *FCRLA* associate with HIV-1 vaccine protection in the RV144 trial and other similar studies and across individual immune cell subsets. Also, it would be useful to re-evaluate the same RV144 ALVAC-HIV-1 plus gp120 HIV-1 vaccine regimen in animal models like humanized mice [24] and non-human primates, especially by matching animals with the corresponding genotypes described here and in [2]. This would firmly recapitulate the relevance of these polymorphisms in conferring protection, and offer new avenues for designing an improved vaccine regimen.

## Supporting Information

S1 Fig *FCGR2C* polymorphisms associate with FcγR gene expression in B cells from all 462 individuals. Similar as Fig 1, but added with individuals from the Yoruba population in red triangles.
(PDF)

S2 Fig Overview of the associations of SNPs with the expression of *FCGR2A*. Part of the FcγR region (~182 kb) is shown in the UCSC genome browser (hg19, chr1:161,468,905–161,651,105). The top track shows the genomic locations and the association p-values (-log10 scale) for those SNPs that passed the significance cutoff of FDR < 0.05 in their associations with the expression of *FCGR2A* at different levels as reported in [7], i.e. exon, transcript, and gene. Three vertical bands in color cyan highlight the three SNPs with the most significant p-values (S1 Table and also see S2 Table), where the left-most one covers the SNPs described in [2]. The middle two horizontal tracks show the LD between each of the corresponding SNPs shown on top track and the SNP rs114945036. The last two tracks show the gene annotations from RefSeq and Gencode (basic annotation set). Two green vertical bands highlight the last exon of *FCGR2A* and *FCGR2C*.
(PDF)

S3 Fig The expression of *FCGR2A* and *FCGR2C* is highly correlated in human B cells. A. Scatterplot of raw RNA-seq read counts (log2 scale) of RefSeq annotated *FCGR2C* (x-axis) and *FCGR2A* (y-axis) in B cells from each of 462 individuals. The number on the top-right corner shows the Pearson correlation coefficient. B. Similar as A, but limited to the expression of the last exons of both genes.
(PDF)

S1 Table The eQTLs with the most significant p-values of the association between SNPs and the expression of *FCGR2A* or *FCRLA* identified in human B cells in the EUR population. (XLSX)

S2 Table List of significant eQTLs for FCGR2A and FCRLA mapped within the Fc region (XLSX)

S3 Table Summary of transcript sequence similarities between *FCGR2A* and *FCGR2C*. (XLSX)
