# Supplementary figures and images for: FCGR2C Polymorphisms Associated with HIV-1 Vaccine Protection Are Linked to Altered Gene Expression of Fc-γ Receptors in Human B Cells
Source: PLoS One. 2016 Mar 25;11(3):e0152425. doi: 10.1371/journal.pone.0152425 (PMC4807760; doi:10.1371/journal.pone.0152425)

# Supplemental Figure 1

A

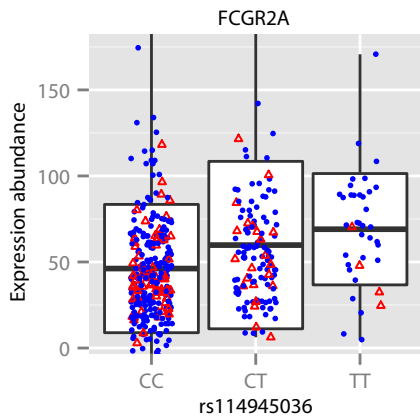

B

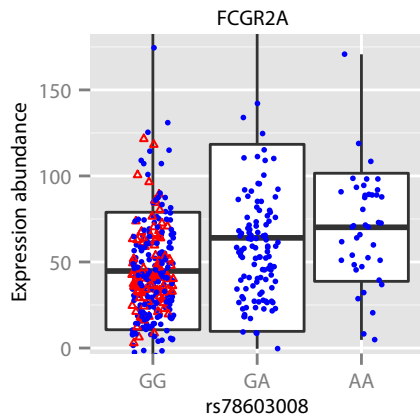

C

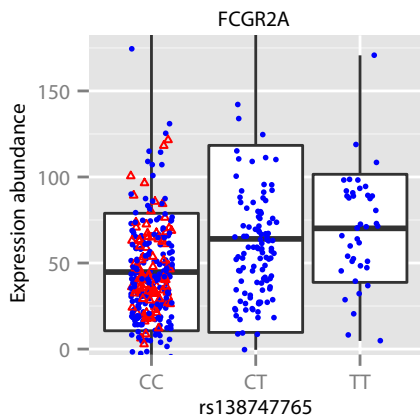

D

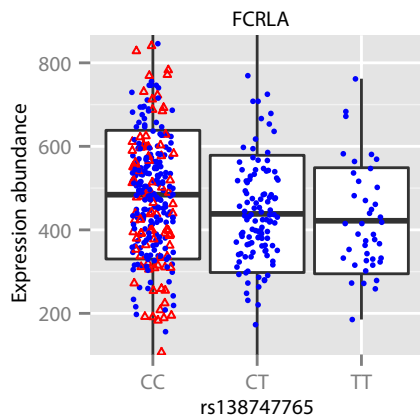

Supplement: S1 Fig — Similar as Fig 1, but added with individuals from the Yoruba population in red triangles. (PDF) [file pone.0152425.s001.pdf]

## Supplemental Figure 2

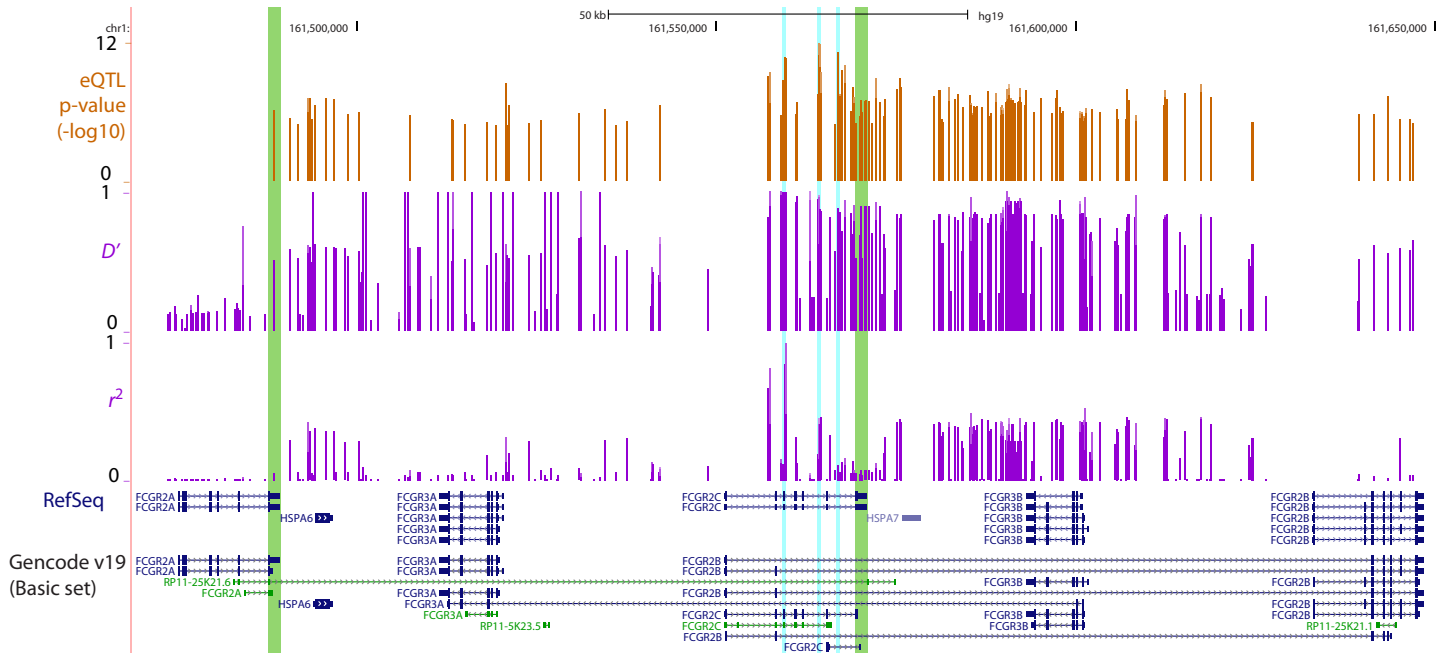

Supplement: S2 Fig — Part of the FcγR region (~182 kb) is shown in the UCSC genome browser (hg19, chr1:161,468,905–161,651,105). The top track shows the genomic locations and the association p-values (-log10 scale) for those SNPs that passed the significance cutoff of FDR < 0.05 in their associations with the expression of FCGR2A at different levels as reported in [7], i.e. exon, transcript, and gene. Three vertical bands in color cyan highlight the three SNPs with the most significant p-values (S1 Table and also see S2 Table), where the left-most one covers the SNPs described in [2]. The middle two horizontal tracks show the LD between each of the corresponding SNPs shown on top track and the SNP rs114945036. The last two tracks show the gene annotations from RefSeq and Gencode (basic annotation set). Two green vertical bands highlight the last exon of FCGR2A and FCGR2C. (PDF) [file pone.0152425.s002.pdf]

# Supplemental Figure 3

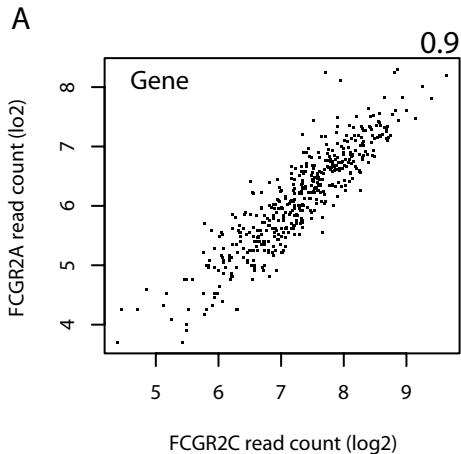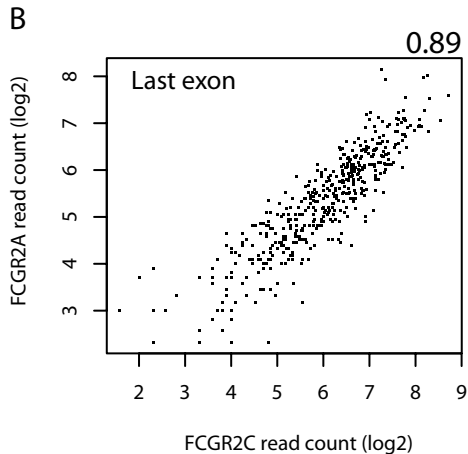

Supplement: S3 Fig — A. Scatterplot of raw RNA-seq read counts (log2 scale) of RefSeq annotated FCGR2C (x-axis) and FCGR2A (y-axis) in B cells from each of 462 individuals. The number on the top-right corner shows the Pearson correlation coefficient. B. Similar as A, but limited to the expression of the last exons of both genes. (PDF) [file pone.0152425.s003.pdf]
